# P-1298. A Comparison of Daptomycin and Linezolid in Treating Intraabdominal Infections Complicated by VRE Bacteremia

**DOI:** 10.1093/ofid/ofaf695.1486

**Published:** 2026-01-11

**Authors:** Tsung-Yu Tsai, Yu-Chung Chuang, Jia-Ling Yang, Jann-Tay Wang, Yee-Chun Chen, Shan-Chwen Chang

**Affiliations:** Department of Internal Medicine, National Taiwan University Hospital, Taipei, Taiwan, Taipei, Taipei, Taiwan (Republic of China); National Taiwan University Hospital and National Taiwan University College of Medicine, Taipei, Taipei, Taiwan; Department of Internal Medicine, National Taiwan University Hospital, Taipei, Taiwan, Taipei, Taipei, Taiwan (Republic of China); Division of Internal Medicine, National Taiwan University Hospital, Taipei, Taiwan, Taipei, Taipei, Taiwan; National Taiwan University Hospital, Taipei, Taipei, Taiwan (Republic of China); Division of Internal Medicine, National Taiwan University Hospital, Taipei, Taiwan, Taipei, Taipei, Taiwan

## Abstract

**Background:**

Vancomycin-resistant Enterococci (VRE) are pathogenic in intraabdominal infections (IAIs). While daptomycin (8–12 mg/kg) is effective for VRE bloodstream infections (BSIs), evidence supporting its use in VRE-IAIs remains limited. This study aims to evaluate the clinical efficacy of daptomycin versus linezolid in treating VRE-IAIs with BSI.

**Table 1:**
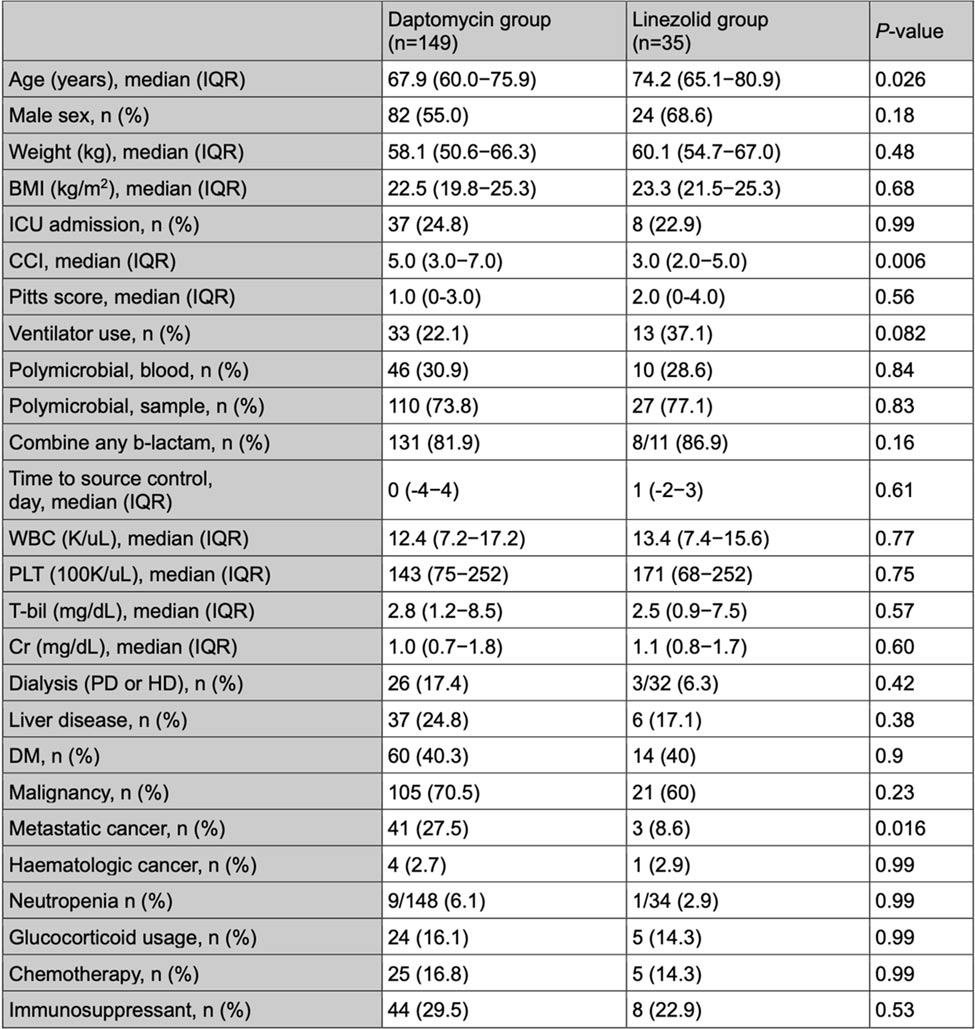
The baseline characteristics of the patients in the daptomycin group and the linezolid group.

**Figure 1:**
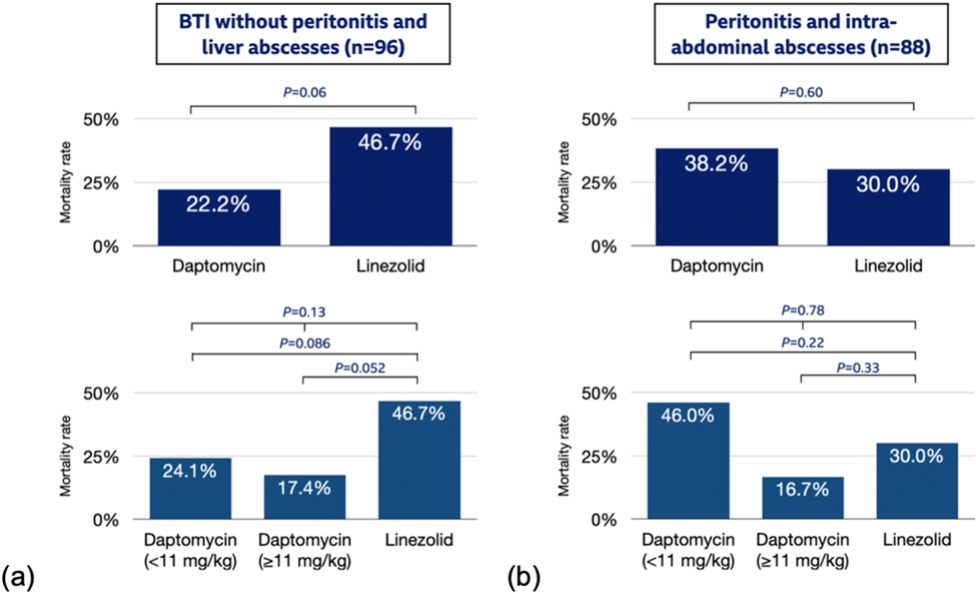
The mortality rate of patients with (a) BTI without peritonitis and liver abscesses (n=96) and with (b) peritonitis and intraabdominal abscesses (n=88), separated by the usage of linezolid, high-dose daptomycin, and low-dose daptomycin.

**Methods:**

We conducted a multicenter observational study. From April 2010 to October 2024, VRE-BSI patients with IAIs as the infection source treated with daptomycin or linezolid were included. The primary outcome was the 28-day in-hospital mortality.

**Figure 2.**
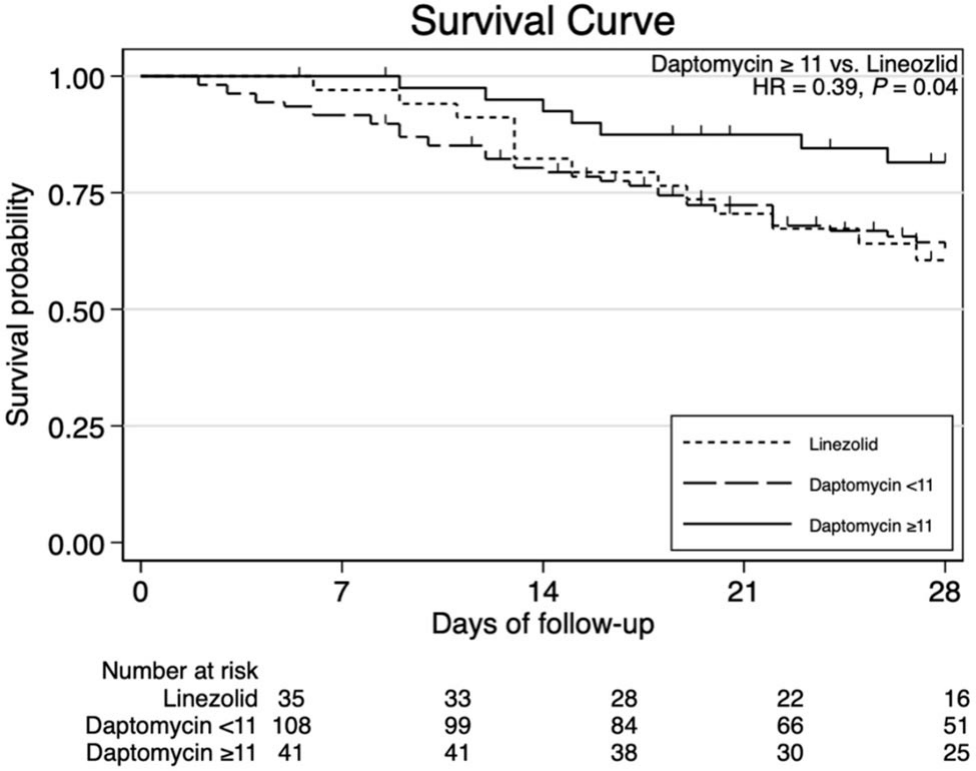
The Kaplan–Meier curve of patients received linezolid, high-dose daptomycin (≥11 mg/kg), and low-dose daptomycin (<11 mg/kg).

**Results:**

Of 184 patients included, 149 received daptomycin and 35 received linezolid (Table 1). Most patients (98.3%) underwent source control procedures. The 28-day mortality did not differ significantly between daptomycin and linezolid groups (29.5% vs. 37.1%; P=0.38). The infection types included biliary tract infections (BTIs) in 75 patients (40.8%), peritonitis in 74 (40.2%), liver abscesses or infected cysts in 21 (11.4%), and intraabdominal abscesses in 14 (7.6%). Multivariable logistic regression identified the independent predictors of mortality including higher Pitt Bacteremia Score (adjusted odds ratio [aOR], 1.22; 95% confidence interval [CI], 1.06–1.41; *P*=0.006), lower platelet count (aOR, 0.996; 95% CI, 0.99–1.00; *P*=0.023), and malignancy (aOR, 2.41; 95% CI, 1.10–5.25; *P*=0.0027). Daptomycin was not associated with increased mortality versus linezolid (aOR, 0.63; 95% CI, 0.28–1.44; *P*=0.27). However, high-dose daptomycin (≥11 mg/kg) was linked to reduced mortality (aOR, 0.29; 95% CI, 0.09–0.91; *P*=0.03), whereas low-dose daptomycin showed no significant difference (aOR, 0.79; 95% CI, 0.34–1.85; *P*=0.59). Regimen changes due to adverse events were similar between the high- and low-dose daptomycin groups (1.89% vs. 2.44%; *P*=0.99). Mortality by infection types and survival curves are shown in Figures 1 and 2.

**Conclusion:**

Daptomycin appears to be at least as effective as linezolid for the treatment of VRE-IAIs with BSI. Moreover, High-dose daptomycin (≥11 mg/kg) was related to improved survival. Further studies in VRE-IAIs without concurrent BSI are needed to clarify daptomycin’s effectiveness.

**Disclosures:**

All Authors: No reported disclosures

